# Long-Term Survival and Curative-Intent Treatment in Hepatitis B or C Virus-Associated Hepatocellular Carcinoma Patients Diagnosed during Screening

**DOI:** 10.3390/biology11111597

**Published:** 2022-11-01

**Authors:** Francesco Izzo, Meredith C. Mason, Eric J. Silberfein, Nader N. Massarweh, Cary Hsu, Hop S. Tran Cao, Raffaele Palaia, Mauro Piccirillo, Andrea Belli, Renato Patrone, Roberta Fusco, Vincenza Granata, Steven A. Curley

**Affiliations:** 1 Department of Surgical Oncology, IRCCS Fondazione “G. Pascale” National Cancer Institute, 80131 Naples, Italy; 2 Department of Surgery, Baylor College of Medicine, Houston, TX 77030, USA; 3 Surgical and Perioperative Care, Atlanta VA Health Care System, Decatur, GA 30033, USA; 4 Division of Surgical Oncology, Department of Surgery, Emory University School of Medicine, Atlanta, GA 30307, USA; 5 Department of Surgery, Morehouse School of Medicine, Atlanta, GA 30310, USA; 6 Medical Oncolody Division, Igea SpA, 80013 Naples, Italy; 7 Italian Society of Medical and Interventional Radiology (SIRM), SIRM Foundation, via della Signora 2, 20122 Milan, Italy; 8 Division of Radiology, Istituto Nazionale Tumori IRCCS Fondazione Pascale—IRCCS di Napoli, 80131 Naples, Italy; 9 Oncology Institute, Christus Trinity Mother Frances Health System, Tyler, TX 75702, USA

**Keywords:** hepatocellular carcinoma, screening, early stage, survival

## Abstract

**Simple Summary:**

This study supports the utilization of active and ongoing HCC screening in high-risk populations with chronic viral hepatitis and cirrhosis. Although we describe curative-intent treatments in 70% of those diagnosed with HCC, more than double that of previous studies, the 10-year survival rate is only 4% due to progressive cirrhosis and HCC recurrence. This study highlights the need for the prevention of cirrhosis and subsequent hepatocarcinogenesis. Improvement in neoadjuvant and adjuvant therapies for HCC merits further investigation. Future work is also needed to measure HCC risk reduction with new effective hepatitis virus treatments.

**Abstract:**

Background: We initiated a prospective screening trial in patients with hepatitis to diagnose HCC in the early stage and to evaluate the impact on long-term survival. Methods: From 1993–2006, 10,372 patients with chronic hepatitis B (14%), hepatitis C (81%), or both (5%) were enrolled in an HCC screening program. All patients underwent liver biopsy at enrollment. Transabdominal ultrasonography and serum alpha-fetoprotein were evaluated every 6 months. Abnormal screening results led to axial imaging and tumor biopsy. Results: Cirrhosis was confirmed on biopsy in 2074 patients (20%). HCC was diagnosed in 1016 patients (9.8%), all of whom had cirrhosis (49.0% HCC incidence in patients with cirrhosis). HCC was diagnosed at the initial screening in 165 patients (16.2%) and on follow-up in 851 patients (83.8%). The HCC diagnosis median time during follow-up screening was 6 years (range 4–10). Curative-intent treatment (resection, ablation, or transplant) was performed in 713 patients (70.2%). Overall survival at 5 and 10 years in those 713 patients was 30% and 4%, respectively, compared to no 5-year survivors in the 303 patients with advanced-stage disease (*p* < 0.001). Cause of death at 5 years in the 713 patients treated with curative intent was HCC in 371 patients (52%), progressive cirrhosis in 116 patients (16%), and other causes in 14 patients (2%). At 10 years, 456 patients (64%) had died from HCC, 171 (24%) from progressive cirrhosis, and 57 (8%) from other causes. Conclusions: Our screening program diagnosed early-stage HCC, permitting curative-intent treatment in 70%, but the 10-year survival rate is 4% due to HCC recurrence and progressive cirrhosis.

## 1. Introduction

Nearly 800,000 new cases and over 740,000 deaths from hepatocellular carcinoma (HCC) occur worldwide each year, making it the second leading cause of cancer-related mortality across the world [1]. Unlike many malignancies that are strongly associated with hereditary or acquired genetic factors, HCC most often develops sporadically as a result of genetic and epigenetic events directly attributable to exposure-related risk factors [2]. The most common contributing risk factor for HCC is the development of cirrhosis from chronic infection with the hepatitis B virus (HBV) and/or the hepatitis C virus (HCV) [3,4,5]. The progression from initial HBV and/or HCV infection to chronic hepatitis and to cirrhosis ultimately leads to hepatocarcinogenesis and the development of HCC [2,6,7,8]. Limiting the progression to cirrhosis in patients with hepatitis virus infections requires the early identification and initiation of effective anti-viral treatment. Similarly, detecting early-stage, potentially curable HCC depends on the recognition of high-risk patient populations and the development of cost-effective diagnostic strategies.

Despite the wide prevalence and increasing number of new cases of HCC annually, HCC is often asymptomatic, and curative treatment options are limited due to late-stage disease at initial diagnosis [9]. Potentially curative therapies include thermal tumor ablation, liver resection, and transplantation [10,11,12,13]. With the exception of liver transplantation, curative-intent options are only appropriate for early-stage disease isolated to the liver in patients with reasonably well-preserved livers—representing only 10% to 30% of all HCC patients at initial presentation [14]. Based on our prior work, we hypothesize that the earlier patients are diagnosed, the more likely they are to have the American Joint Committee on Cancer (AJCC) T1N0M0 or T2N0M0 disease, affording the opportunity to be considered for curative-intent treatment [15]. The patients for whom screening would be most effective are patients with chronic HBV, HCV, or both, as they are at the highest risk of cirrhosis and subsequent hepatocarcinogenesis.

Although the benefit of screening programs for the detection of disease is not in question, what remains unknown is the long-term impact of such screening programs. Specifically, the ability of large, prospective screening trials to diagnose HCC at early stages has been suggested, but the long-term survival benefit and cost-effectiveness are unclear [16]. Additionally, the impact of earlier diagnosis and the delivery of curative-intent treatment on long-term survival is unknown. Using this updated, long-term prospective HCC screening trial based on our prior work [15,17] the goals of this study are to ascertain whether earlier-stage HCC is diagnosed in high-risk patients with HBV and/or HCV and evaluate long-term survival in a cohort of patients with a complete 10-year follow-up.

## 2. Methods

### 2.1. Study Cohort

#### Patient Selection

The Local Ethics Committee approved this prospective multi-center study. All research was conducted in accordance with the Declarations of Helsinki.

According to the study protocol, between 1993 and 2006, patients from the Campania region of Italy voluntarily enrolled in a prospective HCC screening program. Consent was obtained from all enrolled patients.

At enrollment, all patients had a serologic diagnosis of HBV and/or HCV, had a liver biopsy within a year of enrollment to document the baseline severity of liver injury for each patient (including the presence of cirrhosis), and were concurrently undergoing regular clinical examinations.

Patients were excluded from the study if they did not have chronic viral hepatitis infections or a complete follow-up of almost 10 years.

### 2.2. Screening Methods

At the time of enrollment, a serum alpha-fetoprotein (AFP) measurement and an initial transabdominal ultrasound (US) were obtained for each patient. The serum AFP and US were repeated and reassessed every six months during the study period.

In patients for whom a new tumor was detected upon US scan and/or an elevated AFP measurement was documented (defined as >10 ng/mL) at any point during the course of the study, further cross-sectional imaging of the abdomen with either computed tomography (CT) or magnetic resonance imaging (MRI) was obtained, the modality based on the patient’s MR compatibility.

For CT, the study protocol included baseline examination and post-contrast assessment during the arterial, portal, and late phase of the contrast study using a contrast agent with an iodine concentration of at least 370 mg.

The MR study protocol included conventional sequences (T1 and T2 weighted sequences) and functional sequences (diffusion-weighted imaging and post-contrast sequences during the arterial, portal, and late or transitional phases of the contrast study). According to the radiologist’s decision, due to clinical questions, either hepatospecific or interstitial contrast media were employed.

Biopsy by percutaneous fine needle aspiration (FNA) was performed in patients with CT or MRI results suspicious for HCC. If the FNA biopsy results were equivocal for a pathologic diagnosis of HCC, a core needle biopsy was performed.

### 2.3. Treatment

For patients with confirmed HCC on imaging and biopsy at any point during the study period, decisions regarding treatment modality and timing were based on the degree of underlying liver injury and on the stage of HCC. Treatment was initiated according to multi-disciplinary team decision.

According to international guidelines [9], tumor ablation, surgical liver resection, or liver transplantation is recommended and utilized as the curative-intent procedure for early-stage disease. In particular, ablation techniques included both percutaneous radiofrequency ablation (RFA) and percutaneous ethanol injection (PEI) during the first three years of the study. Thereafter, the RFA-only technique was used up to 2009, followed by both RFA and PEI, and RFA and microwave ablation (MWA) after 2009 [9].

For patients with later-stage disease and/or more severe underlying liver injuries who required palliative rather than curative-intent therapy, treatment options included trans-arterial embolization (TAE), trans-arterial chemoembolization (TACE), radio-embolization (from 2008 forward), systemic chemotherapy with sorafenib, and/or supportive care [9].

### 2.4. Statistical Analysis

The Kruskal–Wallis test was utilized to analyze differences in patient characteristics (age, size of tumor, AFP, etc.). Differences between treatment groups with respect to tumor burden, local recurrence, and lesions on follow-up were determined using chi-squared or Fisher’s exact tests. Overall survival (OS) was estimated using the Kaplan–Meier method, and survival was compared between groups using the log-rank test. Statistical significance for all analyses was defined using a *p*-value of <0.05. The Statistics Toolbox of MATLAB R2007a (MathWorks, Inc., Natick, MA, USA) was used to perform all analyses for this study.

## 3. Results

### 3.1. Baseline Clinical Characteristics

A total of 10,372 patients were enrolled and followed prospectively. The baseline patient and disease characteristics are summarized in Table 1. Notably, the majority of patients in the cohort were male (69.6%), had HCV infection alone (81.0%), and had mild chronic active hepatitis (54.0%) on initial liver biopsy. Of the 10,732 patients, 2074 (20.0%) had biopsy-proven cirrhosis.

The screening program detected 1016 patients with HCC, the majority of whom (851 patients (83.7%)) were diagnosed on follow-up screening at some point over the study period. The median time to the diagnosis of HCC in these 851 patients was 6 years (range 4–10 years). Of the 1016 patients with HCC, 100% also had biopsy-proven cirrhosis (Table 2). The rate of false-positive findings using the screening strategy was 4.6%. Most patients with HCC were classified as having either Child–Pugh class A (36.2%) or B (51.3%) cirrhosis.

### 3.2. HCC Treatment

Table 3 describes the treatment details of the 713 patients treated with curative-intent treatment, stratified by Child–Pugh class.

Liver resection was performed on 311 patients (43.6%), the majority of whom were classified as Child–Pugh class A. The patients who were classified as Child–Pugh class B or C were initially medically optimized and subsequently underwent no more than a single segment resection or non-anatomic wedge resection, based on the judgment of the operating surgeon.

Ablation techniques were utilized in 365 patients (51.2%), the majority of whom were Child–Pugh class B. Orthotopic liver transplantation was performed in 37 patients (5.2%).

The remaining 303 patients with HCC not suitable for curative-intent treatment were treated with palliative measures. These patients were diagnosed with advanced-stage disease, including major vascular invasion or extra-hepatic metastatic disease. Of those 303 patients, 237 (78.2%) received some type of therapy (TAE, TACE, radio-embolization, or systemic sorafenib).

Of the remaining 66 patients that received supportive care only, 57 (86.3%) were classified as Child–Pugh B or C. The treatment details in accordance with the BCLC staging system of all 1016 patients diagnosed with HCC within the screening program are reported in Table 4.

### 3.3. Overall Survival in HCC Patients

Of the 1016 patients diagnosed with HCC, the 5-year OS rate was 20% and the 10-year OS rate was 4% (Table 5).

The patients with earlier-stage disease who received curative-intent treatments (713 patients) demonstrated significantly better OS compared to the more advanced-stage patients who received only palliative measures (5-year OS: 30.0% curative-intent vs. 0% palliative; *p* < 0.001, ANOVA). Overall survival at ten years remains at 4% in patients who received curative-intent treatment.

**Figure 1:**
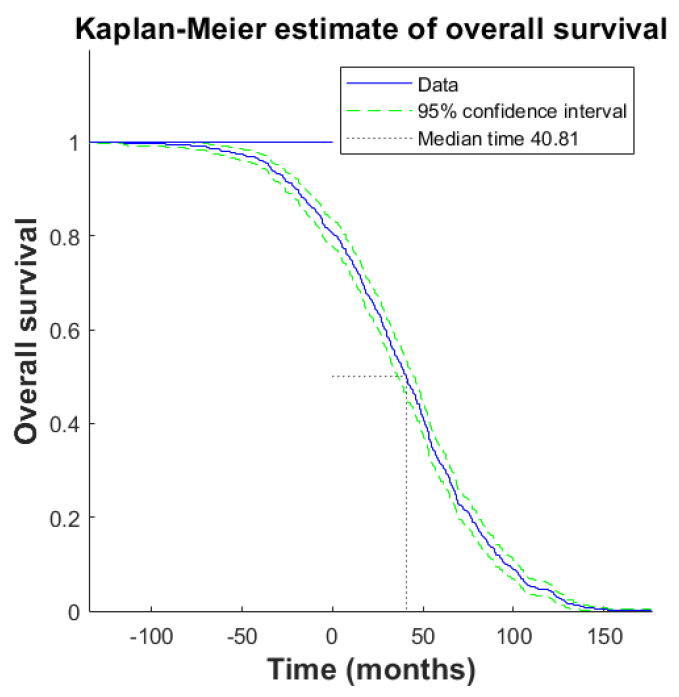
Overall survival function estimation in hepatocellular cancer (HCC) patients who received curative-intent surgical or tumor ablation treatment.

The overall survival function estimation is plotted in Figure 1 with a median time of 40.8 months.

Of patients with cirrhosis without HCC at enrollment who then developed HCC at subsequent follow-up screening (851 patients), Child–Pugh class A patients demonstrated significantly better (log-rank, *p* < 0.01) 5-year survival (28%) compared to Child–Pugh B (15%) and Child–Pugh C (12%) patients, irrespective of the type of curative-intent treatment they received. Additionally, the total number of liver tumors present at HCC diagnosis was associated with OS. The patients with a single tumor had a 5-year OS of 34%, whereas patients with two or more tumors had a 5-year OS of 20% (log-rank, *p* < 0.01). Furthermore, when HCC patients were compared based on their viral etiologies, HCC patients with HBV only had better 5-year OS (23%) than HCC patients with HCV only (16%; log-rank, *p* < 0.05).

Cause of death at 5 years in the 713 patients treated with curative intent was HCC in 371 (52%), progressive cirrhosis in 116 (16%), and other causes in 14 (2%). At 10 years, 456 patients (64%) had died from HCC, 171 (24%) from progressive cirrhosis, and 57 (8%) from other causes. Notably, of the 39 patients who died from cirrhosis within one year of treatment, 15 (38.5%) died from liver failure within 90 days of curative-intent treatment, yielding a treatment-related mortality rate of 2.1%.

## 4. Discussion

Although the utility of a prospective HCC screening has been acknowledged, the impact of such a screening program on early detection and survival in a large, at-risk population has not been previously described. This updated report of over 10,000 patients with a complete 10-year follow-up yields several important findings. All patients who were diagnosed with HCC through this screening program had underlying cirrhosis. Liver parenchymal assessment was performed on a routine hematoxylin–eosin stain, combined with a trichrome or reticulin stain for assessment of fibrosis. Grading and staging were simplified into three grades of necroinflammatory activity and three stages of fibrosis, respectively. The three grades of necroinflammatory activity are “inactive” (portal inflammation only or rare foci of interface or lobular hepatitis; no confluent necrosis), “active, nonsevere” (varying degrees of interface and lobular hepatitis easily identified at low power; no confluent necrosis), and “active, severe” (confluent necrosis, perivenular dropout, bridging necrosis, or parenchymal collapse). The three stages of fibrosis are “early” (no fibrosis or portal fibrosis), “intermediate” (fibrous septa, focal or frequent), and “advanced” (fibrous septa with focal or diffuse nodularity). The progressive patterns are broad fibrous septa with loosely aggregated collagen fibers, edema, congestion, and inflammatory cells. In progressive disease, the inflammatory activity is initially marked but gradually decreases and is masked by the activation of macrophages and hepatic stellate cells, increasing the deposition of collagen with broad fibrous septa formation and the appearance of ductular reactions.

Of the patients screened and diagnosed with HCC, over 80% were diagnosed at some point during the ongoing follow-up, with a median time to diagnosis of six years. Early-stage disease was detected in 70% of these HCC patients, allowing for curative-intent treatments to be performed. Finally, despite the early detection of HCC in the majority of patients during this screening program, which permitted curative-intent treatments and significantly better overall survival at five years, longer-term overall survival remains poor, with only 4% alive at ten years.

The goal of any screening program is to detect malignant disease at an early stage, when potentially curative treatments may be offered. In the case of breast and colorectal cancers, for which the general incidence and prevalence are much higher, age-appropriate inclusive screening of the whole population is both cost-effective and beneficial for overall survival [18,19]. However, in HCC, which primarily affects patients with cirrhosis (from underlying viral hepatitis infection, some other environmental toxin exposure, metabolic disease, or steatohepatitis), screening the entire population without identifying those with risk factors predisposing to an increased probability of developing HCC would lead to wasteful spending and potentially unnecessary and harmful procedures. However, targeting an at-risk population is feasible, demonstrates a low false-positive rate, and provides a way to detect potentially curable diseases [20,21,22]. Whereas previous screening studies have reported that 30% or less of HCC patients were diagnosed with potentially curable disease [14,23], our study successfully diagnosed 70% of the total HCC patients identified during screening with early-stage disease, allowing curative-intent treatment to be offered.

Once patients have progressed from chronic hepatitis to cirrhosis, mortality increases significantly. One-year survival by Child–Pugh class (newly diagnosed cirrhosis with no HCC) has been estimated at 100% for Child–Pugh A and 80% for Child–Pugh B, dropping to 45% for Child–Pugh C [24]. In cirrhotic patients who undergo abdominal surgery, mortality is 10% for Child–Pugh A and 30% for Child–Pugh B, increasing to >80% for Child–Pugh C [25]. Patients with HCC and cirrhosis have two potentially lethal diseases. At the full 10-year follow-up, 64% of patients in this study had died from HCC and 24% had died from progressive cirrhosis, which underscores the importance of a screening program and the need to diagnose HCC and hepatitis virus infection early, while curative and preventative treatment options are available. Relating to prevention, anti-viral therapies such as ledipasvir/sofosbuvir have demonstrated >95% HCV cure rates in non-cirrhotic patients [26,27], a promising advancement considering that progression to cirrhosis from chronic HCV is a major risk factor for the development of HCC [3,4,5].

For those who do develop HCC, there is still a lack of effective neoadjuvant and adjuvant chemotherapy, targeted therapy, and immunotherapy agents for HCC treated with curative-intent surgical and tumor ablation approaches [28]. HCC is generally refractory to chemotherapeutic agents as a result of high drug-resistance gene expression [29]. Studies using doxorubicin as a neoadjuvant therapy for HCC prior to liver transplant showed a meager overall response to treatment of less than 20% [30]. Furthermore, the survival advantage for those who did respond to neoadjuvant chemotherapy was only a few weeks beyond the best supportive care [30]. Additionally, there are few studies that have shown some survival benefits associated with the administration of adjuvant chemotherapy following curative-intent surgery [31], but these studies are small and not randomized [32,33,34]. A recent meta-analysis of systemic therapies based on 10,100 studies, including 22,113 patients undergoing adjuvant (n = 7) and primary treatment for early (n = 2), intermediate (n = 7), and advanced (first-line, n = 21; second-line, n = 12) stages of disease, suggested that immunotherapies may be more effective in HCC with viral etiologies [35]. Future larger randomized studies are needed to investigate novel neoadjuvant and adjuvant systemic therapies for HCC to improve the long-term survival rates of patients treated with curative intent [36,37,38,39,40,41,42,43,44].

Several important limitations of this study should be considered when interpreting our findings. Because the entire cohort was composed of Caucasian/Italian patients, the overall generalizability to the overall population is limited, and therefore, our results should be interpreted accordingly. Importantly, comprehensive details of the patient comorbidity burden, including other chronic medical conditions and alcohol intake, were not available for the cohort, and, therefore, these factors could not be taken into account with respect to our survival results. Our survival analyses were grouped by all curative-intent treatments versus all palliative treatments as the overall sample size was not large enough to conduct individual analyses by treatment type separately. Furthermore, the choice of treatment modality was not randomized or matched as this was a descriptive study and it was not designed or powered to detect subtle differences in treatment-specific outcomes.

Another limitation is related to the fact that we did not assess non-alcoholic fatty liver disease (NAFLD) and its complications in our study. Since there is an increasing NAFLD clinical significance, particularly in Western nations, due to a rising burden of metabolic syndrome, screening programs in this subgroup would also be appropriate. In fact, although a prior retrospective analysis has shown that the cumulative incidence of HCC is slightly lower in NAFLD-related cirrhosis compared to HCV cirrhosis (2.6% vs. 4%), surveillance is still recommended in this population. A very low incidence of HCC has been described in patients with NAFLD without cirrhosis; however, incidence rates do not meet surveillance criteria at this time [37,38]. Continued investigation of these relationships is of utmost importance, given the increasing prevalence and incidence of NAFLD [45,46,47,48].

## 5. Conclusions

This study supports the utilization of active and ongoing HCC screening in high-risk populations with chronic viral hepatitis and cirrhosis. Although we describe curative-intent treatments in 70% of those diagnosed with HCC, more than double that of previous studies, the 10-year survival rate is only 4% due to progressive cirrhosis and HCC recurrence. This study highlights the need for the prevention of cirrhosis and subsequent hepatocarcinogenesis. Improvement in neoadjuvant and adjuvant therapies for HCC merits further investigation. Future work is also needed to measure HCC risk reduction with new effective hepatitis virus treatments.

## Figures and Tables

**Table 1:** Patient and clinical characteristics in patients with chronic hepatitis B and/or C virus infection screened for hepatocellular cancer (HCC) (N = 10,372).

Patient Characteristics	N = 10,372
Age, years, mean SD (range)	61.2 ± 9.3 (25–86)
Male sex, no. (%)	7219 (69.6%)
Caucasian race	10,372 (100%)
**Disease Characteristics**	
Hepatitis B infection, no. (%)	1452 (14.0%)
Hepatitis C infection, no. (%)	8401 (81.0%)
Hepatitis B + C infection, no. (%)	518 (5.0%)
**Hepatic parenchyma**	
Mild chronic active hepatitis, no. (%)	5601 (54.0%)
Severe chronic active hepatitis, no. (%)	2697 (26.0%)
Cirrhosis, n.o. (%)	2074 (20.0%)
**Malignancy**	
Diagnosed HCC, no. (%)	1016 (9.8%)
Diagnosed on initial screening	165 (1.6%)
Diagnosed on follow-up screening	851 (8.2%)

**Table 2:** Characteristics of the 1016 patients diagnosed with hepatocellular cancer (HCC) among a screening population of 10,372 chronic hepatitis B and/or C virus-infected individuals.

Patients with HCC	n = 1016
Age, years, mean SD (range)	60.0 ± 9.8 (28.0–80.0)
HCC, diagnosed on initial screening, n (%)	165 (16.2%)
HCC, diagnosed on follow-up screening, n (%) *	851 (83.8%)
Tumor size, cm, mean SD (range)	4.1 (2.2–15.0)
Number of tumors, n, mean SD (range)	2 (1–6)
AFP, ng/mL, mean SD (range)	269 (91–3200)
Biopsy-proven cirrhosis, n (%)	1016 (100%)
Child–Pugh class	
A, n (%)	368 (36.2%)
B, n (%)	521 (51.3%)
C, n (%)	127 (12.5%)
**Stage in curative-intent patients ^#^**	**n = 713**
I	530 (74.3%)
II	183 (25.7%)
III	0
IV	0

* For patients diagnosed during follow-up, the interval time was 4 to 10 years. **^#^** Patients are classified using the TNM staging system for HCC.

**Table 3:** Treatment demographics by Child–Pugh class in the 713 patients with hepatocellular cancer (HCC) treated with curative-intent treatment.

Treatment Type	Child’s A (n = 289, 40.5%)	Child’s B (n = 326, 45.7%)	Child’s C (n = 98, 13.7%)	Total (n = 713)
Liver resection, n (%)	182 (63.0%)	125 (38.3%)	4 (4.1%)	311 (43.6%)
Ablation (RFA, MWA, PEI), n (%)	102 (35.3%)	189 (58.0%)	74 (75.5%)	365 (51.2%)
Liver transplantation, n (%)	5 (1.7%)	12 (3.7%)	20 (20.4%)	37 (5.2%)

**Table 4:** Treatment demographics by BCLC stage in the 1016 patients with hepatocellular cancer (HCC) treated with both curative and non-curative-intent treatments.

Treatment Type	BCLC Stage 0/A (n = 479, 47.1%)	BCLC Stage B (n = 252, 24.8%)	BCLC Stage C (n = 121, 11.9 %)	BCLC Stage D (n = 164, 16.1%)
Liver resection, n (%)	268 (55.9%)	26 (10.3%)	13 (10.7%)	4 (2.4%)
Ablation (RFA, MWA, PEI), n (%)	211 (44.1%)	58 (23.1%)	22 (18.2%)	74 (45.1%)
Liver transplantation, n (%)	0	17 (6.7%)	0	20 (12.2%)
Palliative treatments (TAE, TACE, radio-embolization, or systemic therapy)	0	151 (59.9%)	86 (71.1%)	0
Supportive care	0	0	0	66 (40.2%)

**Table 5:** Overall survival in hepatocellular cancer (HCC) patients who received curative-intent surgical or tumor ablation treatment.

Status	Early-Stage Patients Receiving Curative-Intent Treatment (n = 713)	
	5-Year	10-Year
Death from HCC	371 (52.0%)	456 (64.0%)
Death from cirrhosis complications	115 (16.1%)	171 (24.0%)
Death from other causes	14 (2.0%)	57 (8.0%)
Overall survival rate	213 (29.9%)	29 (4.1%)

## Data Availability

Data are reported at the link https://zenodo.org/record/7266551#.Y2pj_3bMK3B (accessed on 10 September 2022).
